# 2196. Frequencies of Adenovirus Types in U.S. Children with Acute Respiratory Illness, 2016–2019

**DOI:** 10.1093/ofid/ofac492.1815

**Published:** 2022-12-15

**Authors:** Varvara Probst, Tess Stopczynski, Justin Z Amarin, Andrew J Spieker, Herdi Kurnia Rahman, Laura S Stewart, Rangaraj Selvarangan, Jennifer E Schuster, Marian G Michaels, John Williams, Julie A Boom, Leila C Sahni, Vasanthi Avadhanula, Mary A Staat, Elizabeth P Schlaudecker, Monica McNeal, Christopher J Harrison, Mary E Moffatt, Geoffrey A Weinberg, Peter G Szilagyi, Janet A Englund, Eileen J Klein, Aaron T Curns, Ariana Perez, Benjamin R Clopper, Brian Rha, Susan I Gerber, James Chappell, Natasha B Halasa

**Affiliations:** Vanderbilt Univerisity Medical Center, Nashville, Tennessee; Vanderbilt University Medical Center, Nashville, Tennessee; Vanderbilt University Medical Center, Nashville, Tennessee; Vanderbilt University, Nashville, Tennessee; Vanderbilt University Medical Center, Nashville, Tennessee; Vanderbilt University Medical Center, Nashville, Tennessee; Children's Mercy, Leawood, Kansas; Children's Mercy Kansas City, Kansas City, Missouri; University of Pittsburgh School of Medicine, UPMC Children’s Hospital of Pittsburgh, Pittsburgh, PA; UPMC Children's Hospital of Pittsburgh, Pittsburgh, Pennsylvania; Baylor College of Medicine, Houston, Texas; Baylor College of Medicine, Texas Children’s Hospital, Houston, Texas; Baylor College of Medicine, Houston, Texas; CCHMC, Cincinnati, Ohio; University of Cincinnati College of Medicine, Cincinnati, Ohio; Cincinnati Children's Hospital Medical Center, Cincinnati, Ohio; Children's Mercy - Kansas City, Kansas City, Missouri; Children's Mercy Kansas City, University of Missouri Kansas City School of Medicine, Kansas City, Missouri; University of Rochester/UR-Golisano Children's Hosp, Rochester, New York; University of Rochester School of Medicine and Dentistry, Rochester, New York; Seattle Children's Hospital/ Univ. Washington, Seattle, Washington; University of Washington/Seattle Children's Hospital, Seattle, Washington; Centers for Disease Control and Prevention, Atlanta, Georgia; CDC, Atlanta, Georgia; US Centers for Disease Control and Prevention, Atlanta, Georgia; Division of Viral Diseases, U.S. Centers for Disease Control and Prevention, Atlanta, Georgia; US CDC, Atlanta, Georgia; Vanderbilt University Medical Center, Nashville, Tennessee; Vanderbilt University Medical Center, Nashville, Tennessee

## Abstract

**Background:**

Adenovirus (AdV) is a common cause of acute respiratory illness (ARI). Multiple respiratory AdV types have been identified in humans, but it remains unclear which are the most common in U.S. children with ARI.

**Methods:**

We conducted a multicenter, prospective viral surveillance study at seven U.S. children’s hospitals, the New Vaccine Surveillance Network, during 12/1/16–11/30/19, prior to the COVID-19 pandemic. Children < 18 years of age seen in the emergency department or hospitalized with fever and/or respiratory symptoms were enrolled, and mid-turbinate nasal +/- throat swabs were tested using multiplex respiratory pathogen assays or real time polymerase chain reaction (PCR) test for AdV, respiratory syncytial virus (RSV), human metapneumovirus, rhinovirus/enterovirus (RV), influenza, parainfluenza viruses, and endemic coronaviruses. AdV-positive specimens were subsequently typed using single-plex qPCR assays targeting sequences in the hexon gene specific for types 1-7, 11, 14, 16 and 21. Demographics, clinical characteristics, and outcomes were compared between AdV types.

**Results:**

Of 29,381 enrolled children, 2,106 (7.2%) tested positive for AdV. The distribution of types among the 1,330 (63.2%) successfully typed specimens were as follows: 31.7% AdV-2, 28.9% AdV-1, 15.3% AdV-3, 7.9% AdV-5, 5.9% AdV-7, 1.4% AdV-4, 1.2% AdV-6, 0.5% AdV-14, 0.2% AdV-21, 0.1% AdV-11, and 7.0% ≥1 AdV type. Most children with AdV-1 or AdV-2 detection were < 5 years of age (**Figure 1a**). Demographic and clinical characteristics varied by AdV types, including age, race/ethnicity, smoke exposure, daycare/school attendance, and hospitalization (**Table 1**). Co-detection with other viruses was common among all AdV types, with RV and RSV being the most frequently co-detected (**Figure 1b**). Fever and cough were the most common symptoms for all AdV types (**Figure 2**). Children with AdV-7 detected as single pathogen had higher odds of hospitalization (adjusted odds ratio 6.34 [95% CI: 3.10, 12.95], p= 0.027).

**Figure:**
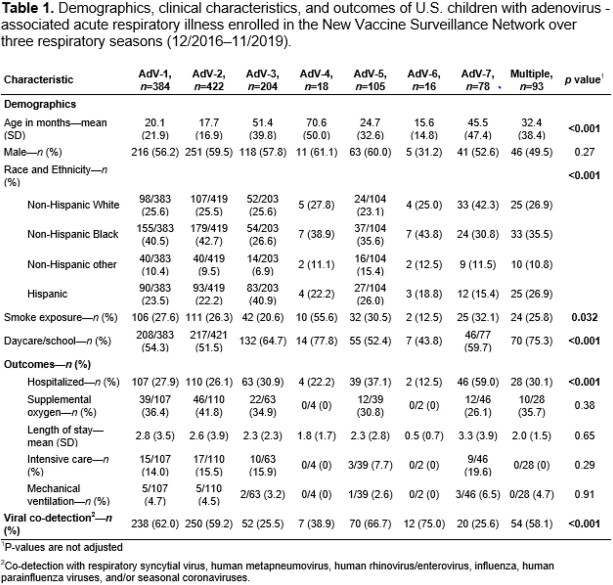

**Figure:**
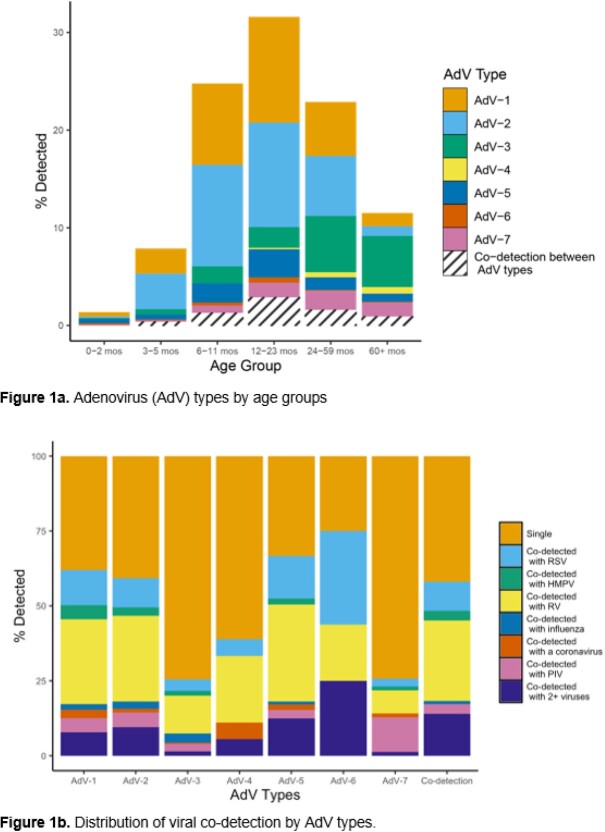

**Figure:**
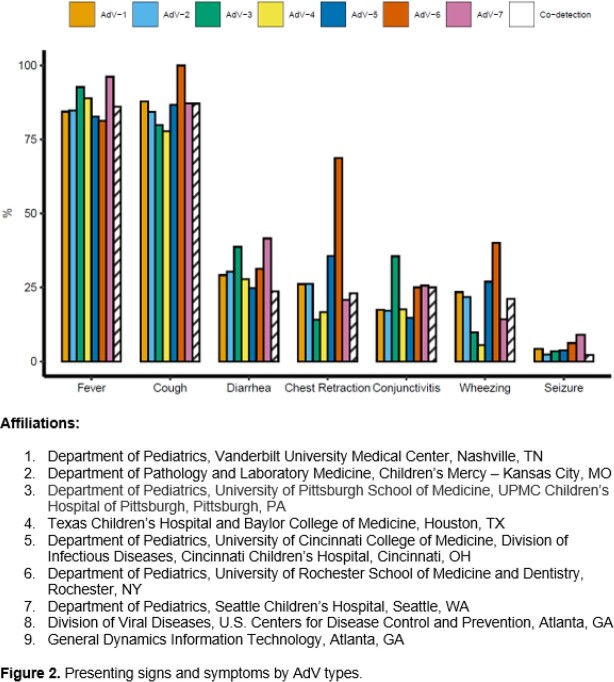

**Conclusion:**

AdV-2 and AdV-1 were the most frequently detected AdV types among children over the 3-year study period. Notable clinical heterogeneity of the AdV types warrants further surveillance studies to identify AdV types that could be targeted for pediatric vaccine development.

**Disclosures:**

**Rangaraj Selvarangan, BVSc, PhD, D(ABMM), FIDSA, F(AAM)**, BioFire: Grant/Research Support|Luminex: Grant/Research Support **John Williams, MD**, GlaxoSmithKline: Advisor/Consultant|Quidel: Advisor/Consultant **Mary A. Staat, MD, MPH**, Centers for Disease Control and Prevention: Grant/Research Support|Cepheid: Grant/Research Support|National Institute of Health: Grant/Research Support|Uptodate: Royalties **Christopher J Harrison, MD**, Astellas: Grant/Research Support|GSK: Grant/Research Support|Merck: Grant/Research Support|Pediatric news: Honoraria|Pfizer: Grant/Research Support **Mary E. Moffatt, M.D.**, Becton and Dickinson and Company: Stocks/Bonds|Biogen: Stocks/Bonds|Coloplast B: Stocks/Bonds|Express Scripts: Stocks/Bonds|Novo Nordisk A/S Spons ADR: Stocks/Bonds|Novo Nordisk A/S-B: Stocks/Bonds|Steris PLC: Stocks/Bonds|Stryker Corp: Stocks/Bonds|Thermo Fisher Scientific: Stocks/Bonds **Geoffrey A. Weinberg, MD**, Merck & Co.: Honoraria|Merck & Co.: Honoraria for composing and reviewing textbook chapters, Merck Manual of Therapeutics **Janet A. Englund, MD**, AstraZeneca: Advisor/Consultant|AstraZeneca: Grant/Research Support|GlaxoSmithKline: Grant/Research Support|Meissa Vaccines: Advisor/Consultant|Merck: Grant/Research Support|Pfizer: Grant/Research Support|Sanofi Pasteur: Advisor/Consultant **Natasha B. Halasa, MD**, Quidel: Grant/Research Support|Quidel: equipment donation|Sanofi: Grant/Research Support|Sanofi: HAI testing and vaccine donation.

